# Lipid peptide nanocomplexes for gene delivery and magnetic resonance imaging in the brain

**DOI:** 10.1016/j.jconrel.2012.07.002

**Published:** 2012-09-10

**Authors:** Michele J. Writer, Panagiotis G. Kyrtatos, Alison S. Bienemann, John A. Pugh, Andrew S. Lowe, Claudio Villegas-Llerena, Gavin D. Kenny, Edward A. White, Steven S. Gill, Cameron W. McLeod, Mark F. Lythgoe, Stephen L. Hart

**Affiliations:** a Molecular Immunology Unit, UCL Institute of Child Health, London WC1N 1EH, UK; b Centre for Advanced Biomedical Imaging, Department of Medicine and UCL Institute of Child Health, University College London, London WC1E 6DD, UK; c The Functional Neurosurgery Research Group, Bristol University, Institute of Clinical Neurosciences, Southmead Hospital, Bristol BS16 1LE, UK; d Centre For Analytical Sciences, University of Sheffield, Sheffield S10 2TN, UK

**Keywords:** Nanoparticle, Gene delivery, MRI, Brain, Targeting

## Abstract

Gadolinium-labelled nanocomplexes offer prospects for the development of real-time, non-invasive imaging strategies to visualise the location of gene delivery by MRI. In this study, targeted nanoparticle formulations were prepared comprising a cationic liposome (L) containing a Gd-chelated lipid at 10, 15 and 20% by weight of total lipid, a receptor-targeted, DNA-binding peptide (P) and plasmid DNA (D), which electrostatically self-assembled into LPD nanocomplexes. The LPD formulation containing the liposome with 15% Gd-chelated lipid displayed optimal peptide-targeted, transfection efficiency. MRI conspicuity peaked at 4 h after incubation of the nanocomplexes with cells, suggesting enhancement by cellular uptake and trafficking. This was supported by time course confocal microscopy analysis of transfections with fluorescently-labelled LPD nanocomplexes. Gd-LPD nanocomplexes delivered to rat brains by convection-enhanced delivery were visible by MRI at 6 h, 24 h and 48 h after administration. Histological brain sections analysed by laser ablation-inductively coupled plasma-mass spectrometry (LA-ICP-MS) confirmed that the MRI signal was associated with the distribution of Gd^3 +^ moieties and differentiated MRI signals due to haemorrhage. The transfected brain cells near the injection site appeared to be mostly microglial. This study shows the potential of Gd-LPD nanocomplexes for simultaneous delivery of contrast agents and genes for real-time monitoring of gene therapy in the brain.

## Graphical abstract

**Figure:**
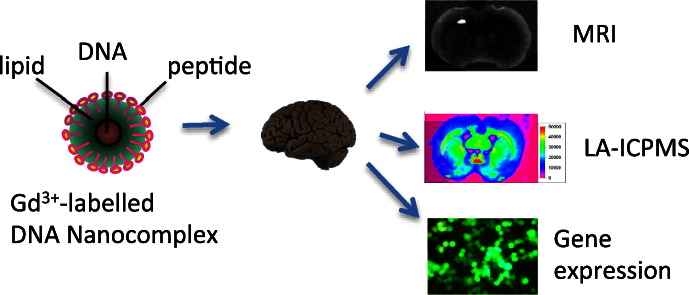

## 1 Introduction

Nucleic acid therapeutics offer opportunities for the development of new medicines for a diverse range of unmet clinical needs in the brain including neurodegenerative diseases, neuromuscular diseases, and gliomas. Synthetic nanoparticle formulations, such as lipoplexes and polyplexes, with a variety of chemical formulations are being investigated for the delivery of therapeutic genes and siRNAs to the brain [1,2]. Although systemic routes of delivery to the brain are an attractive option for some formulations [3] for others delivery is often inefficient since the blood–brain barrier is highly selective and may exclude high molecular weight nanoparticles [4]. Methods of direct administration of nanoparticles to the brain, such as convection-enhanced delivery (CED), physically bypass the BBB and can achieve widespread dispersal of therapeutics from a single infusion with appropriate types of nanoparticles [5–7].

Not only is delivery to the brain problematic but also the inaccessibility of the brain for sampling and monitoring of treatment presents further challenges. Magnetic resonance imaging (MRI) provides a high resolution non-invasive tool to monitor brain pathologies and potential treatment regimes using agents that affect image contrast. T_1_ agents such as Gd are particularly useful as they produce a ‘bright’ spot on MRl whereas T_2_ iron-containing contrast agents produce dark areas and can suffer from problems of discriminating adjacent areas with the same hypointensities. Generally, contrast agents for MRI offer limited sensitivity and so packaging of the Gd^3 +^ contrast agents into nanoparticles, such as liposomes or polymer based nanoparticles has been investigated as a means to concentrate the agents and enhance their specific delivery to the tissues of interest [8,9]. Thus, the properties of different nanoparticle formulations, such as tissue targeting and cellular uptake can be exploited to further enhance the diversity of possible biomedical applications of MRI [10].

We have described previously a formulation of liposomes and receptor-targeted, cationic peptides, which self-assemble on mixing with DNA or siRNA into nanocomplexes and display efficient, targeted transfection [11]. In this study we have investigated the potential of these multifunctional nanocomplexes for imaging of distribution by MRI *in vitro* and *in vivo* in rat brains. Nanocomplexes were labelled with Gd^3 +^-chelated lipids formulated into the liposome component at a range of concentrations (10–20% Gd^3 +^) and assessed in transfections of cultured cells to determine their MRI characteristics, transfection efficiency and toxicity. Then optimised complexes were injected into rat brains and analysed by MRI and fluorescent immunohistochemistry to localise both vector distribution, indicated by Gd^3 +^, and GFP reporter gene expression. MRI distribution data was further validated by LA-ICP-MS analysis of tissue sections.

## 2 Materials and methods

### 2.1 Cell lines

U87-MG human glioblastoma cells (ECACC, Porton Down, Wilts) were maintained in Eagles MEM with Earle's balanced salt solution containing 2 mM l-glutamine, 1% non-essential amino acids, 1 mM sodium pyruvate, 10% FCS and antibiotic–antimycotic (Invitrogen, Paisley, UK).

### 2.2 Lipids and peptides

1,2-Dioleoyl-3-(trimethylammonio)propane (DOTAP) and 1,2-dioleoyl-*sn*-glycero-3-phosphoethanolamine-N-(lissamine rhodamine B sulfonyl) (ammonium salt) were obtained from Avanti Polar lipids (Alabaster, AL, USA). Dioleoylphosphatidylethanolamine (DOPE) and diethylenetriaminepentaacetic acid α,ω-bis(8-stearoylamido-3,6-dioxaoctylamide) gadolinium salt (C_62_H_116_N_7_O_14_Gd; M_*r*_ 1340.87) (Gd-DTPA-DSOA), [12], were obtained from Sigma Aldrich (Poole, UK). Peptide P (K_16_GACLPHKSMPCG) and the control with a scrambled targeting motif, peptide PS, (K_16_GACHPPMSKLCG) were synthesised by Alta Biosciences (Birmingham, UK).

### 2.3 Liposome formulations and MRI analysis

Lipids were dissolved in chloroform at 10 mg/ml and mixed to give formulations with a range of Gd-lipid content (0–20% of total lipid) (Table 1) followed by production of a lipid film by rotary evaporation at 40 °C. Lipids were rehydrated with sterile, distilled water preheated to 40 °C to give a final concentration of 1 mg/ml. Liposomes were incubated at 4 °C overnight followed by sonication in a water bath. MR imaging of liposome MW6 (Table 1) was performed on a 9.4 T VNMRS horizontal bore (Agilent, Palo Alto, USA) using a 59/33 quadrature volume coil (Rapid, Würzburg, Germany), with the dilution series placed into a Perspex holder within the RF coil. The longitudinal relaxivity r_1_ was determined from a linear fit of 1/T_1_ as a function of Gd^3 +^ concentration.

### 2.4 Nanocomplex formation, biophysical analysis and MRI

Complexes were prepared by mixing components in the order lipid:peptide:DNA (L:P:D) in the weight ratios of 1:4:1 (unless otherwise stated) which corresponds to a free nitrogen to phosphate (N/P) ratio of approximately + 5. Formulations were prepared in OptiMEM for cell culture, or in water for MRI and biophysical analysis, as described previously [13]. The average diameter and zeta potential of the nanocomplexes were determined using a Nano S Zetasizer (Malvern Instruments, Malvern, UK) at a temperature of 25 °C, viscosity of 0.89 cP and a refractive index of 1.33. The z-average value was determined from ten size measurements on three occasions. From this analysis the polydispersity index (PI) was obtained to determine sample homogeneity with a value of > 0.3 indicating a heterogeneous population. The zeta potentials of nanocomplexes were determined from their electrophoretic mobilities in an electric field in the Nano S Zetasizer.

LD and LPD complexes for MRI analysis were prepared in water to a total volume of 125 μl as follows: LD: 20 μg lipid in 87.5 μl water added to 20 μg DNA in 37.5 μl water, LPD (lipid, peptide and DNA): 20 μg lipid in 37.5 μl water, 80 μg peptide in 50 μl followed by 20 μg DNA in 37.5 μl. Complexes were mixed by pipetting up and down after the addition of the DNA and allowed to stand at room temperature for 30 min before scanning.

### 2.5 Transfections and gene expression analysis

Cells were plated (10,000 per well) in 24-well plates then the transfection complex formulated in OptiMEM was added in 200 μl per well containing 250 ng of the luciferase-expressing plasmid pCI-Luc. Plates were incubated for 4 h at 37 °C then replaced with complete medium and incubated for a further 24 h at 37 °C before preparing cell lysates and measuring luciferase gene expression by luminometry by the kit instructions (Promega, Southampton, UK). Luminescence as relative light units (RLU) was measured on an Optima Fluostar microplate reader (BMG Labtech, Aylesbury, UK) then the protein concentration was measured using a Bio-Rad protein assay (Hemel Hempstead, UK) and RLU values standardised to protein values (RLU/mg). Cell viability assays were performed with the CellTiter 96 Aqueous One Solution Cell Proliferation Assay (Promega, Southampton, UK).

### 2.6 Transfections for MRI analysis

U87-MG glioblastoma cells were plated at 0.5 × 10^6^ cells per well in 6-well plates then transfected as above but with complexes containing 1 μg pCI-Luc plasmid in 1.5 ml OptiMEM. For image analysis by MRI, cells were trypsinised then pelleted and fixed for 20 min in ice-cold, 1% paraformaldehyde-phosphate buffered saline (PBS), before pelleting again and resuspending the pellet in 270 μl PBS in a polypropylene tube and repelleting.

### 2.7 Magnetic resonance imaging of nanocomplexes and transfected cells

LPD nanocomplexes (MW6:P:pEGFP) were prepared in water as described above. MRI measurements were performed at room temperature on a 9.4 T VNMRS horizontal bore scanner (Varian Inc. Palo Alto, CA) with a shielded gradient system (400 mT/m); 59/33 Rapid quadrature volume coil. A T_1_ weighted spin echo sequence was acquired for qualitative evaluation (matrix 384 × 192; field of view 80 × 40 mm; TR = 500 ms; TE = 19.4 ms; NSA = 2; slc = 0.5 mm). For quantitative maps either an inversion recovery prepped spin echo sequence was acquired (matrix 256 × 92; FOV 8 × 40 mm; TR = 16,500 ms; TE = 16.9 ms; NSA = 1; TI = 100, 300, 500, 700, 900 and 16,000 ms) or a saturation recovery (512 × 256 matrix, slc = 0.5 mm, NSA = 4, FOV 60 × 30 mm, TE = 10.9 ms at TR = 250–5000 ms). T_1_ maps were calculated on a pixel wise basis. The mean (± standard deviation) T_1_ relaxation time of individual cell pellets was obtained by manually contouring a region of interest around each pellet.

### 2.8 Confocal fluorescence microscopy

1.5 × 10^5^ U87-MG cells were seeded on 35 mm FluoroDishes (World Precision Instruments Inc., FL, USA) in complete medium. After 24 h the cells were transfected with nanoparticles in OptiMEM formulated as described previously with: pCI-Luc DNA labelled with Cy5 as per manufacturer's protocol (Kreatech, Amsterdam, Netherlands), peptide P and MW6:DOPE-rhodamine (99:1 wt/wt) at a 1:4:1 weight ratio. Transfections were performed by adding complexes to wells (0.5 μg pCILuc per well). After 1, 2, 4 and 6 h of incubation, the cells were washed with PBS and fixed in 4% formaldehyde, permeabilised with 0.2% Triton, blocked with 1% BSA and stained for 20 min with AlexaFluor 488 phalloidin (1:50, or 4U/ml, Invitrogen, Paisley, UK) and DAPI (0.1 μg/ml, Sigma-Aldrich). The wells were washed and sealed in mounting media (Invitrogen, Paisley, UK) before visualising on a Carl Zeiss LSM710 laser scanning microscope system (Jena, Germany).

### 2.9 *In vivo* infusions

RTN vector formulations were prepared for *in vivo* use in 5% dextrose at a pEGFP DNA concentration of 0.32 mg/ml, and with MW6/DOPE and peptide P at a weight ratio of 1:4:1 (L:P:D). All procedures were carried out in accordance with the UK Home Office regulations. Male Wistar rats weighing 250 g (B&K Universal, Hull, UK) once anaesthetised were placed in a stereotactic frame (Stoelting Co, Wood Dale, Illinois, USA). Burr holes were used to allow cannula implantation to corpus callosum or striatum using the following coordinates: corpus callosum: 1.5 mm anterior and 2.5 mm lateral to the bregma and 2.5 mm ventral; striatum: 0.75 mm anterior and 3.5 mm lateral to the bregma and 4.5 mm ventral. 4 l of MW6:P:pEFGP nanocomplex formulation was infused using a 220 μm outer diameter fused silica cannula connected to a 10 μl Hamilton syringe at a rate of 0.5 μl/min at each site using an infusion pump (World Precision Instruments, Inc., Sarasota, Florida, USA). This corresponded to a dose of 1.3 μg each of the DNA and MW6 lipid (containing 15% Gd-3 + lipid by weight), and 5.2 μg of the peptide. Following infusion, the cannula was left *in situ* for 5 min and withdrawn at a rate of 1 mm/min. Animals were killed 48 h after treatment by transcardial perfusion fixation using 4% paraformaldehyde (pH 7.4) under terminal anaesthesia.

### 2.10 MRI and tissue processing of infused brains

Fixed rat brains were imaged using a T_1_-weighted spin echo sequence (TR = 150 ms, TE = 7 ms, 170 μm resolution, slc = 0.5 mm) and a T_2_*-weighted spoiled gradient echo sequence (TR = 300 ms, TE = 5.5 ms, FA = 60, 85 μm resolution, slc = 0.5 mm). Brain sectioning, and immunofluorescence staining for microglial cells with mouse anti-ED1 (1:100; Serotec, UK) were performed as described previously [14]. All microscopic images were obtained using a Leica DM5500 microscope and digital camera (MBF, Germany). Haematoxylin and eosin staining was performed on sections to examine tissue morphology after nanocomplex infusions.

### 2.11 Analysis of histological sections by laser ablation-inductively coupled plasma mass spectrometry (LA-ICP‐MS)

The laser ablation system (UP-266 Macro LA system, Nd:YAG λ 266 nm, New Wave Research, Cambridgeshire, UK) was configured to perform multiple parallel line rastering to generate elemental (2D) distribution maps. A laser beam diameter of 155 μm was utilised for interrogation of sections. Laser energy was in the range of 1.4 mJ at a frequency of 10 Hz, and the scanning speed was set to 55 μm/s. The interrogated area was in the region of 140 mm^2^. The line rasters were separated by 310 μm, to prevent contamination of adjacent tissue with previous line raster runs. Complete analysis runtime was 178 min. Elemental maps were produced using the Graphis software package (Kylebank Software Ltd., Ayr, UK). The isotopes ^157^Gd and ^57^Fe were monitored in a time-resolved mode using an Agilent 4500 ICP‐MS and were selected on the basis of high-percentage abundance and minimal interferences.

## 3 Results

### 3.1 Biophysical analysis of liposomes and nanocomplexes

Liposomes (L) were formulated with the cationic lipid DOTAP and DOTAP + Gd-DTPA-DSOA (Gd-lipid), at a combined molar ratio relative to DOPE of 1:1 (Table 1). Lipoplexes with pCILuc plasmid DNA at a weight ratio of 1:1 (L:D), and nanocomplexes comprising liposome, peptide and pCILuc plasmid at a weight ratio of 1:4:1 (L:P:D) were formulated (Table 2) in sterile, deionised water. MW6 liposomes (69.7 nm) were similar in size to DOTAP/DOPE liposomes (68.0 nm) while MW5 liposomes were slightly larger (63.5 nm) (*p* < 0.05) and MW7 liposomes (58.5 nm) were slightly smaller (*p* < 0.05) than DOTAP/DOPE liposomes. Nanocomplexes comprising liposomes MW5 (59.3 nm) and MW6 (61.0 nm) formulated with peptide K_16_GACLPHKSMPCG and plasmid pCI-Luc were similar in size to nanocomplexes containing DOTAP/DOPE (58.2 nm) while nanocomplexes with MW7 (71.6 nm) were significantly larger (*p* < 0.05). Size analysis of all four nanocomplexes suggested a single major peak with most particles in each group falling in a size distribution ranging from 30 nm to 100 nm. All polydispersity values were < 0.3 indicating sufficient sample homogeneity for reliability of the measurements.

Based on a mixing ratio of 1:4:1 (L:P:D) and a molecular weight of 3.7 × 10^6^ for pCILuc and 1340.87 for the Gd-lipid, we calculate the number of Gd centres per DNA molecule to be 276 for MW5 nanocomplexes, 414 for MW6 nanocomplexes and 552 for MW7 nanocomplexes. With an estimated 1–5 DNA molecules per nanocomplex based on approximate volume calculations, the maximal numbers of Gd centres per nanocomplex are 1380, 2070 and 2760 for MW5, MW6 and MW7 nanocomplexes respectively.

### 3.2 Transfection efficiency and cytotoxicity of Gd-containing nanocomplexes

**Fig. 1:**
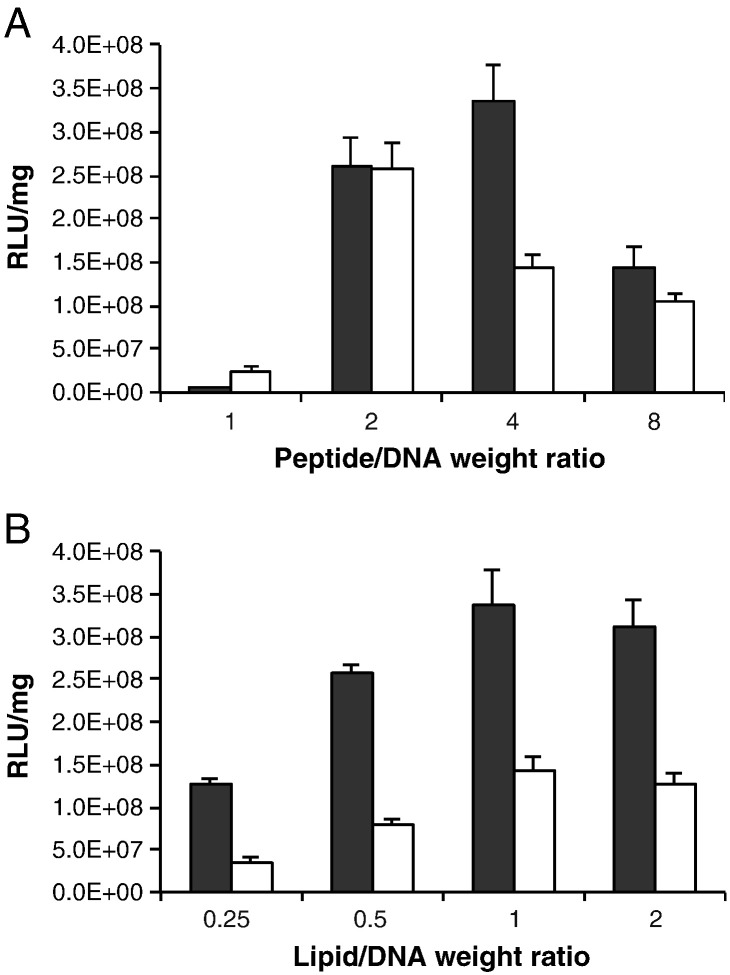
Lipid and peptide optimisation for transfection efficiency and targeting. U87 human glioblastoma cells were transfected with formulations containing either targeting peptide P (black bar) or non-targeting peptide Ps (white bars) with liposome DOTAP/DOPE and plasmid pCI-Luc. Luciferase expression (RLU/mg) was monitored at 48 h post transfection. (A) Peptide:DNA weight ratios were varied in formulations where liposome:DNA was maintained at a constant weight ratio (1:1 L:D). (B) Liposome:DNA weight ratios were varied while peptide:DNA weight ratios remained constant (4:1). The optimal ratios were 1:4:1 and 2:4:1 L:P:D.

LPD formulations were prepared comprising DOTAP/DOPE and either peptide P, or control PS, at a series of weight ratios, varying peptide with constant lipid weight ratio at 1:1 (L:D) (Fig. 1A) then varying lipid with constant peptide (4:1, P:D) (Fig. 1B) and compared for transfection efficiency in U87-MG cells. The optimal lipid ratio was 1:1 (L:D) by weight while the optimal peptide DNA weight ratio was 4:1 w/w. The peptide ratio 4:1 w/w P:D was critical for achieving receptor-mediated transfection. At that ratio the transfection efficiency of peptide P targeted formulations was more than twice that of untargeted, peptide Ps-mediated transfections (Fig. 1A). The transfection efficiency of formulations with a lipid:DNA ratio of 0.5:1 or 0.25:1 L:D w/w was significantly lower than the transfection efficiency at 1:1 although increasing to 2:1 had no significant effect (Fig. 1B). Formulations of LD (no peptide) showed very low levels of transfection (not shown), indicating the functional importance of the peptide component in the LPD mixture.

**Fig. 2:**
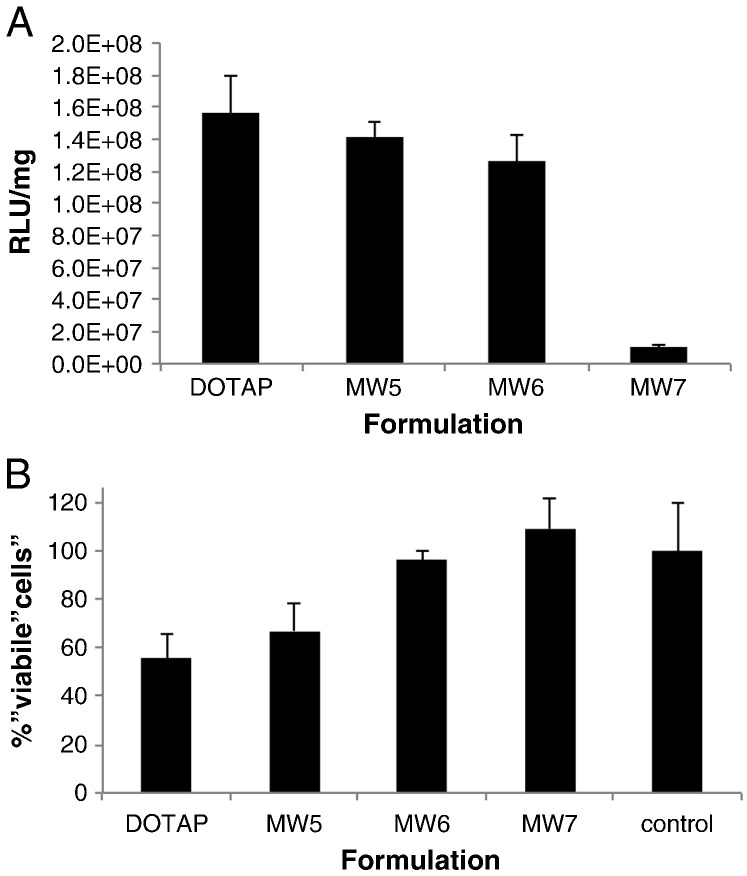
Transfection efficiency of Gd-lipids and cytotoxicity of nanocomplexes. U87-MG cells were transfected with formulations of peptide P and liposomes DOTAP/DOPE, MW5, MW6 or MW7 then analysed by MRI. (A) Nanocomplexes with lipids DOTAP/DOPE, MW5 or MW6 displayed similar levels of luciferase activity (RLU per mg) while MW7 formulations were far less active (*p* < 0.05). (B) A MTT toxicity assay was performed following transfection and cell viability expressed as a percentage of the absorbance at 490 nm obtained from untransfected cells. DOTAP formulations were significantly more toxic than those with MW6 (*p* < 0.05).

The transfection efficiency of LPD nanocomplex formulations containing MW5 or MW6 was similar to that of homologous formulations containing DOTAP while MW7 formulations were approximately 15-fold less efficient than DOTAP/DOPE LPD formulations (Fig. 2A). Viabilities of cells exposed to LPD nanocomplexes containing DOTAP/DOPE were reduced to about 50% while those containing increasing amounts of Gd-DTPA-DSOA displayed a trend for reduced toxicity (Fig. 2B), although this is more likely a consequence of lower cationic DOTAP content (Table 1). Cells transfected with MW6 and MW7 formulations showed no significant loss of viability relative to untransfected cells (*p* > 0.05).

**Fig. 3:**
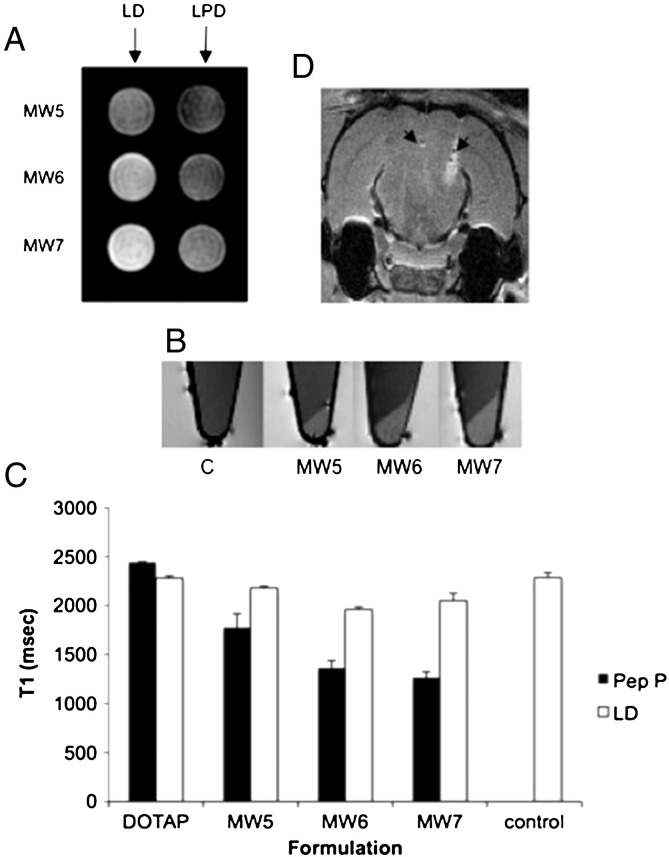
MRI analysis of formulations prepared with plasmid DNA, peptide P and liposome (DOTAP/DOPE, MW5, MW6 or MW7), or lipid:DNA (LD; 1:1 weight ratio) with no peptide. (A) Complexes of LPD or LD were formulated and analysed in microfuge tubes by MRI. LPD nanocomplexes displayed reduced contrast compared to LD formulations. Higher Gd^3 +^ concentration in LD formulations led to increased contrast (MW5 ➔ MW7). (B) U87-MG cells were transfected with nanocomplex formulations and displayed detectable contrast signals compared to untransfected cells (left-most tube). (C) T_1_ values were calculated from MR images of cell pellets transfected with LPD or LD formulations. (D) U87 cells transfected with MW6:P:pCILuc were injected into fixed rat brain and analysed by MRI, to assess the potential for *in vivo* detection.

LD and LPD complexes containing liposomes MW5, MW6 and MW7 were compared in T_1_ measurements of complexes (Fig. 3A). The LPD formulation suspensions displayed longer relaxation times compared to homologous LD complexes, suggesting more limited accessibility of the Gd^3 +^ moiety to water [15] (Fig. 3A). MR images of pelleted cells that had been incubated with MW5, MW6 and MW7 Gd^3 +^‐labelled nanocomplexes for 4 h at 37 °C showed obvious contrast enhancement between cells and medium when compared with the untransfected control cells (Fig. 3B). The relaxation times for the pellets from MW5, MW6 and MW7 transfected cells were significantly lower in LPD formulations compared to LD complexes (Fig. 3C). Relaxation times did not vary significantly between LD formulations containing MW5, MW6 or MW7 whereas LPD formulations showed a significant shortening of T_1_ (p < 0.05) from MW5 to MW6 (Fig. 3C). The increase in Gd^3 +^ content from MW6 (15% total lipid) to MW7 (20% total lipid) did not significantly increase contrast enhancement further. The MW6 lipid appeared to offer the highest Gd^3 +^ content whilst retaining transfection efficiency and so was selected for ongoing MRI studies in cells and *in vivo*. MW6 had an r_1_ value of 2.8 ± 0.02 mM^− 1^ s^− 1^.

To assess the potential for MRI detection of cells in the brain transfected by Gd^3 +^‐containing nanocomplexes, U87-MG cells transfected with MW6-containing LPD formulations were injected into one side of fixed rat brain while a Gd^3 +^‐free control LPD formulation was injected on the other side and imaged. The Gd^3 +^ treated cells were highly conspicuous against the surrounding brain tissue compared to the Gd-free control cells (Fig. 3D).

### 3.3 Kinetics of MRI contrast enhancement

**Fig. 4:**
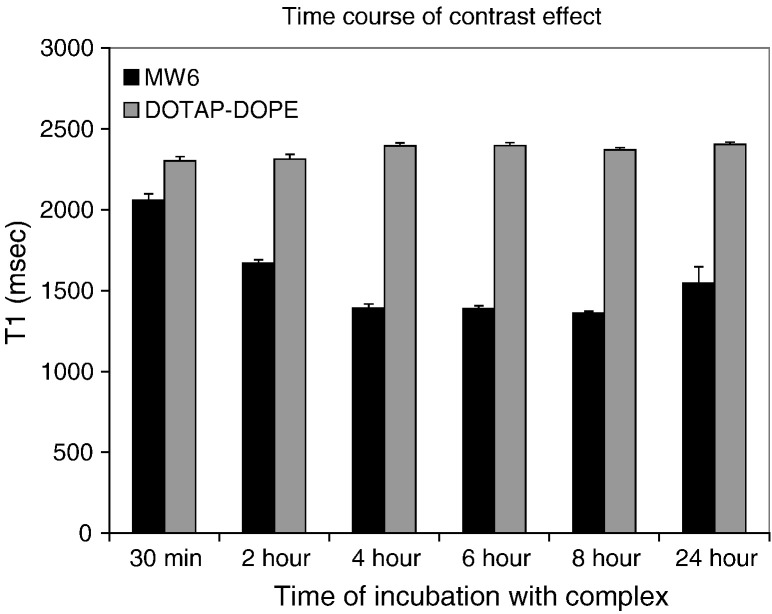
MRI time course *in vitro*. U87-MG cells were transfected with formulations of MW6:P (Gd^3 +^-labelled) or DOTAP/DOPE:P and analysed by MRI. Cells were incubated with complexes for varying lengths of time at 37 °C. At the assay time point, unbound complexes were washed from the cells, the cells harvested, fixed and the cell pellet analysed by MRI (8 h *vs.* 24 h *p* < 0.0005).

LPD nanocomplexes comprising peptide P with MW6 or DOTAP/DOPE were prepared and incubated with U87-MG cells then analysed by MRI at a series of time points from 30 min up to 24 h (Fig. 4). The shortest T_1_ relaxation values were achieved after 4 h of incubation and remained stable at least up to 8 h followed by a gradual increase in relaxation time at 24 h. This time course suggested that the MRI contrast effect was enhanced by cellular uptake of nanocomplexes.

### 3.4 Internalisation of nanocomplexes

**Fig. 5:**
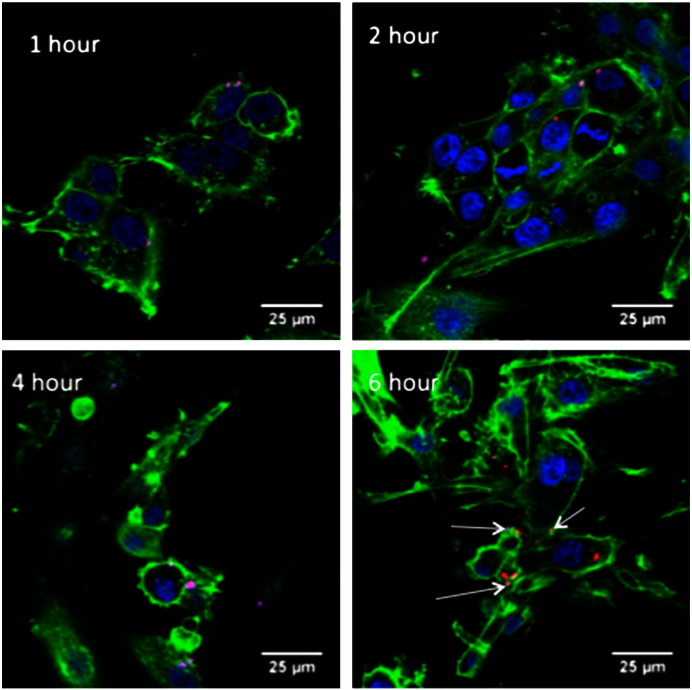
Confocal microscopy images showing cellular uptake of fluorescently labelled LPD nanocomplexes. U87-MG cells were transfected with LPD nanocomplexes containing Cy5-labelled DNA (pink) and rhodamine-labelled liposome (red) and examined at serial time points (1 h, 2 h, 4 h and 6 h). Arrows in 6d highlight Cy5-fluorescence, while other particles are rhodamine.

To investigate the time-course of LPD transfections in cells in terms of nanocomplex uptake and intracellular dissociation, U87-MG cells were transfected with fluorescently-labelled nanocomplexes containing rhodamine-labelled lipid and Cy5-labelled plasmid and analysed by confocal fluorescence microscopy (Fig. 5).

At earlier time points (30 min and 1 h) Cy5 fluorescence was observed predominantly at the cell surface while rhodamine was barely detectable. By 2 h, rhodamine fluorescence was more readily detected, mostly co-localised with Cy5 as punctate staining in the cytoplasm, suggesting endosomal location. Four to 6 h after transfection both fluorophores were more abundantly detected as punctate staining in the cytoplasmic and perinuclear regions. While in many cases they remained co-localised, the fluorophores were detected apart more frequently at this later time point (Fig. 5, 6 h). These observations support the analysis of the MRI kinetics (Fig. 4) suggesting a process of internalisation of intact particles followed by intracellular disassembly and separation of the lipid from the DNA.

### 3.5 MRI, histology and LA-ICP-MS following *in vivo* infusion

The striatum and corpus callosum of the three rat brains were infused by convection enhanced delivery (CED) with LPD nanocomplex formulations containing DOTAP/DOPE or MW6 along with peptide P and plasmid pEGFP at weight ratios of 1:4:1 (L:P:D) with DNA at a concentration of 0.32 mg/ml. Animals were killed at 6 h, 24 h, and 48 h, and the brains were analysed by MRI then sectioned for histological and LA-ICP-MS analysis on adjacent tissue sections.

**Fig. 6:**
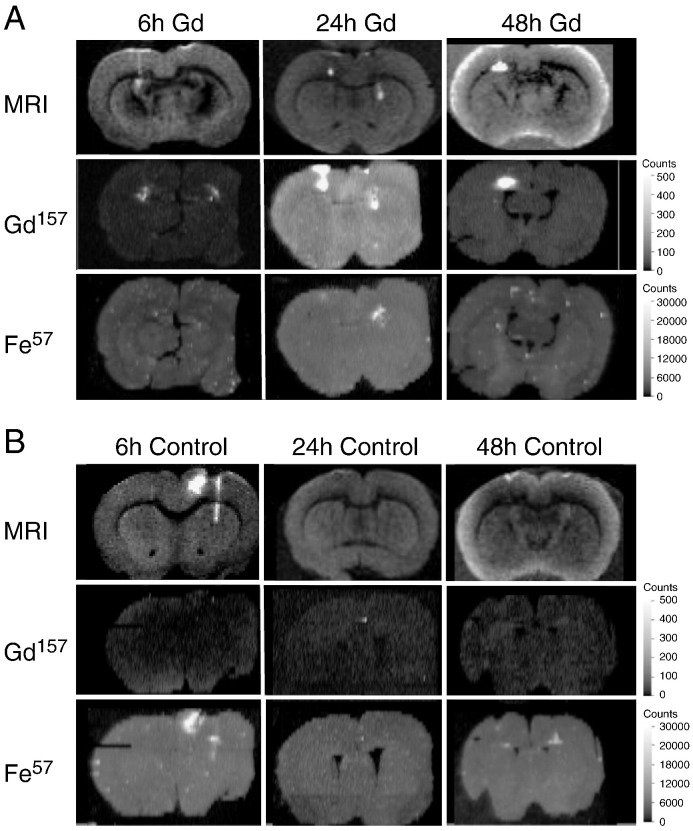
MRI and LA-ICP-MS analysis following *in vivo* infusion. Rats were infused with Gd^3 +^-labelled nanocomplexes (MW6:P) on the left side only (48 h) or both left and right sides (6 h and 24 h). Control rats were infused with Gd^3 +^-free nanocomplexes (DOTAP/DOPE:P) on both left and right sides of the brain. T_1_-weighted MR images and LA-ICP-MS images of ^157^Gd and ^57^Fe distribution of individual rat brains at 6 h, 24 h and 48 h after injection treated with (B) MW6 LPD nanocomplex formulations and (C) DOTAP/DOPE LPD control formulations. The Gd scale for all Gd images was set to 500 counts and the Fe images to 30,000 counts.

MRI analysis revealed a hyperintensity on T_1_-weighted spin-echo images located in the vicinity of the cannula tip in the corpus callosum due to the presence of Gd^3 +^ complexes in the brain parenchyma (Fig. 6A). The control images from rats receiving Gd-free nanocomplexes at 24 h and 48 h after injection did not show any areas of T_1_-weighted hyperintensity (Fig. 6B). However at 6 h a strong MRI signal was observed in the Gd^3 +^-free control. Histologically, this animal presented with signs of haemorrhage at the injection site (data not shown) and iron within haemoglobin is a known potential confounding factor for MRI analysis of Gd^3 +^ distribution [16].

To further investigate the contrast effect observed by MRI, brain tissue sections were analysed by LA-ICP-MS. In Gd^3 +^-treated animals, ^157^Gd distribution maps from all three time points, 6 h, 24 h and 48 h, detected Gd signals coinciding with the MRI contrast (Fig. 6A). The distribution of both MRI and LA-ICP-MS signals remained focal rather than diffuse, even at 48 h, which is consistent with an intracellular distribution of Gd^3 +^ delivered by nanocomplexes. In the Gd^3 +^-treated 6 h and 48 h samples, LA-ICP-MS distribution of ^57^Fe was uniform, indicating that the MRI signal was entirely associated with ^157^Gd (Fig. 6A). However in the 24 h sample the MRI signal detected in the corpus callosum and weakly in the striatum was associated with the detection of a discrete, high-intensity ^57^Fe region, probably arising from small haemorrhages from the injections.

The LA-ICP-MS analysis of control samples treated with Gd^3 +^-free nanocomplexes showed residual background levels (< 100 counts) of ^157^Gd suggesting some minor cross-contamination from previous Gd-enriched samples (Fig. 6B). The 6 h control had a high signal on MRI and histological evidence of haemorrhage; this was confirmed by a very intense ^57^Fe measurement in the ICP‐MS analysis. *i.e.*, Fe was, the likely source of the MRI signal. Iron was also detected (at very low signal) in the 48 h control although there was no change apparent in MRI contrast. It should be noted that the MRI and LA-ICP-MS images at each time point are from individual animals and so it is not possible to say from this experiment whether the differences in MRI and ^157^Gd intensity are significant.

### 3.6 Fluorescence microscopy analysis for GFP and ED-1

**Fig. 7:**
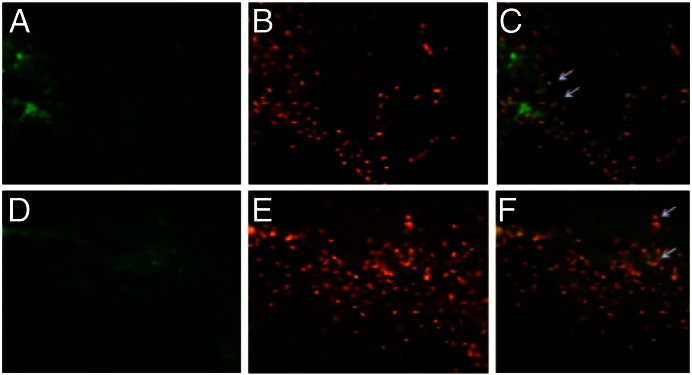
EGFP transfection rat striatum. Rat brains were infused with DOTAP/DOPE:P:pEGFP by CED then histological sections were analysed for localisation of GFP expression. The figure shows representative images of EGFP expression (a and d) detected in the grey matter at 48 h in the presence of ED-1 positive cells (b and e). Colocalisation of positive active microglia was detected within the region highlighted by arrows towards co-localised cells (c and f).

GFP expression was detected by fluorescence microscopy in brain sections 48 h after the rats were infused with the MW6-containing nanocomplexes (Fig. 7a, d). GFP expression was found in the immediate vicinity of the cannula track in the grey matter. Immunostaining identified ED-1-positive microglial cells (Fig. 7b and e). The majority of GFP-expressing cells co-localised with the ED-1 staining (Fig. 7c and f), indicating that the majority of transfected cells were microglial.

## 4 Discussion

The aim of this work was to evaluate a receptor-targeted, Gd^3 +^-labelled nanoparticle formulation for the co-delivery of nucleic acid therapeutics along with contrast agents for detection by MRI in the brain. The potential for brain delivery with long-term expression of transgenes has been reported for a non-viral, nanoparticle formulation [1,2] and the principle of co-delivery of drug and contrast agents for MRI with liposome formulations has been established in previous studies where liposomes were labelled with Gd^3 +^ for co-delivery of drugs to monitor delivery to brain tumours in rats by CED [9] and for real-time imaging of CED of viral gene therapy vectors using a gadolinium contrast agent as a surrogate marker for location of the virus [17]. In this study we aimed to combine these properties into a single formulation for gene transfer and non-invasive detection of vector localisation by MRI.

### 4.1 Gd^3 +^-labelled LPD formulations — biophysical properties

The LPD nanocomplex formulation comprises an electrostatically self-assembling mixture of cationic lipids, peptides and plasmid DNA similar to formulations we have used previously for *in vivo* delivery to lung airways [18], for systemic targeted delivery to tumours [13], and *ex vivo* to vein grafts [19]. The modularity of the lipid and peptide components makes this system very useful for testing new structural variations and delivery modalities. In this study we have used a formulation of a peptide element comprising a cell-surface receptor targeting domain (LPHKSMP), and an oligolysine domain (K_16_) for binding to anionic nucleic acids, separated by a short spacer (—GA-) [20]. The cationic liposome comprised a formulation of cationic DOTAP, and the fusogenic lipid DOPE formulated into liposomes in a 1:1 weight ratio. To enable detection by MRI, the DOTAP/DOPE liposome was substituted for one of a series of alternative Gd^3 +^-labelled liposomes, MW5, MW6 and MW7. In these liposomes a Gd-lipid was substituted for DOTAP so that by weight the Gd-lipid formed 10, 15 or 20% of the liposome formulation, while the DOPE was retained at a constant 50% of total lipid (Table 1). The Gd-lipid is incorporated into the liposome bilayer during the rehydration step of liposome formulation.

The LPD nanocomplex formulations containing DOTAP/DOPE, MW5 and MW6 along with the plasmid DNA and peptide P, were all of similar size, and each significantly smaller than their parent liposomes. LPD nanocomplexes made with MW7 were significantly larger than the other nanocomplexes and the parent MW7 liposome while their transfection efficiencies were considerably lower suggesting that the higher Gd^3 +^ content destabilised the structure of the liposomes and nanocomplexes. The MW6 lipid, containing 15% Gd-lipid offered the optimal combination of highest Gd^3 +^ content while retaining transfection efficiency. The size range of DOTAP, MW5 and MW6 LPD nanocomplexes measured by dynamic light scattering ranged from approximately 30–100 nm and all were highly cationic.

### 4.2 *In vitro* transfections with Gd^3 +^-labelled LPD formulations

The Gd-free LPD formulation DOTAP/DOPE:P:pCILuc transfected U87-MG cells in a receptor-enhanced manner at the optimal weight ratio of L:P:D of 1:4:1. Transfection efficiency was 2-fold better than that of a non-targeted LPD formulation, where binding is completely non-specific due to the net cationic charge of the formulations (N/P ratio of + 5). The receptor targeting peptide LPHKSMP therefore enhanced transfection efficiency rather than providing absolute targeting specificity. To achieve higher degrees of receptor specificity would likely require anionic nanocomplexes to reduce non-specific cell binding. The receptor-mediated enhancement of transfection was only observed at one ratio of peptide:DNA (4:1) while higher and lower ratios of peptide:DNA had a detrimental effect on transfection. This suggests that the nanocomplexes containing peptide at the 4:1 ratio with DNA have the optimal biophysical properties, receptor density and orientation for targeted transfection. The LPHKSMP targeting motif was identified previously by biopanning of a phage peptide library on epithelial cells and it has close homology to the motif LHKSMP, occurring within glycoprotein B of neurotrophic herpes simplex virus which is reported to bind to cell-surface heparan sulphate, although the binding of peptide P to this receptor moiety has not been confirmed [20]. In epithelial cell transfections nanocomplexes containing peptide P displayed a twelve-fold enhancement over non-targeted control formulations whereas in U87-MG cells the enhancement was only two fold suggesting the possibility for improvement of targeted transfection specificity and efficiency with alternative peptide ligand.

Nanocomplexes with 10% (MW5) or 15% Gd-lipid (MW6) incorporated in the liposome bilayer displayed similar transfection efficiencies to nanocomplexes containing Gd-free liposomes suggesting that the lipid function in the transfection pathway such as endosomal fusion [21], was unaffected by the altered lipid ratios or Gd lipid. The transfection efficiency of nanocomplexes with 20% Gd-lipid (MW7) however was significantly compromised. The larger size and less positive zeta potential of the MW7-containing nanocomplexes may explain their lower transfection efficiency, lowering their potential for cell binding and uptake.

The toxicity of nanocomplexes actually decreased with substitution of DOTAP for increasing amounts of the Gd-lipid due most likely to reduced content of DOTAP, as cationic lipids are known to be cytotoxic. The optimal LPD formulation, offering highest Gd^3 +^ content, maximal transfection efficiency and minimal toxicity was therefore liposome MW6 with peptide P.

### 4.3 MRI analysis of *in vitro* transfections

Suspensions of MW6 LPD complexes exhibited lower contrast on T_1_-weighted MR images than LD lipoplexes, (Fig. 3) which is most likely due to the increased particle compaction imparted by the peptide interacting with the DNA reducing accessibility of water to the Gd-moiety in the liposome bilayer. Effects of cell interactions on MRI conspicuity were then evaluated using U87-MG cells, a human glioblastoma cell line as a potential model for the development of tumour therapeutics. MRI analysis of U87-MG cells after transfection with nanocomplexes containing MW6 produced a contrast enhancement between cells and medium and reduced T_1_ relaxation times while LD complexes did not show this effect. A kinetic analysis of T_1_ relaxation in U87-MG cells transfected with the MW6 nanocomplex formulation revealed that after 2 h, T_1_ had decreased and was shortest at 4 h which was maintained up to 24 h when T_1_ increased. We hypothesised that the observed enhancement of MRI conspicuity over the first 4 h of transfection with LPD nanocomplexes reflected their progressive binding, internalisation, endosomal release and subsequent disassembly within the cell, enhancing Gd^3 +^ accessibility to water. By 24 h, Gd^3 +^ content may be becoming diluted by cell division increasing the relaxation times.

We propose that the liposomal components of the LPD nanocomplex contributes to enhanced endosomal escape *via* lipid bilayer fusion processes, as suggested previously [22]. This hypothesis was tested for the P:MW6 formulation by assessing cellular uptake and dissociation of nanocomplexes containing Cy5 plasmid DNA and rhodamine‐labelled lipids by confocal fluorescence microscopy. In the first hour after transfection cell binding and entry occurred, while by 2 h, particles were more apparent in the cytoplasmic region. It was striking in the first hour that while Cy5 was readily detected the rhodamine label was not detectable. This could be due to nanocomplexes remaining tightly compacted and so quenching the rhodamine. By 2–4 h the endocytosed particles appeared to be undergoing disassembly as rhodamine fluorescence became more apparent, although the two fluorophores remained colocalised. However, by 6 h the fluorophores were more frequently detected separately, suggesting that the DNA and lipid had dissociated. Thus the confocal analysis suggests a process and kinetics of Gd-labelled LPD nanocomplex trafficking compatible with the kinetics of MRI contrast enhancement, supporting the proposal above that MRI conspicuity is enhanced by cellular internalisation and complex disassembly. Other lipoplex and polyplex formulations show similar kinetics of cell trafficking and complex dissociation [23].

In contrast, reports of most other liposome-based Gd^3 +^-formulations showed that they are quenched upon intracellular localisation, which was explained as a consequence of endosomal retention where access of Gd^3 +^ in the liposome to water is more restricted [8,24]. In those studies, liposomes rather than lipoplex with plasmid DNA were used which may have different properties and interactions with cells and less able to escape the endosome than LPD formulations.

### 4.4 MRI analysis of *in vivo* transfections

We then investigated the *in vivo* potential of Gd^3 +^-labelled LPD nanocomplexes as contrast agents, performing a pilot study that involved injecting MW6-transfected U87-MG cells into a rat brain stereotactically. Transfected cells were readily detectable by MRI, indicating that cells containing sufficient amounts of the Gd^3 +^-labelled nanocomplex can potentially be detected by MRI *in vivo*. Based on that finding, MW6:P:pEGFP formulations were then injected into the rat striatum or corpus callosum by CED. Brains analysed by MRI revealed a weak signal at 6 h but strong T_1_-weighted signals at 24 h and 48 h after delivery. Similar MRI contrast intensity kinetics were observed *in vivo* in a tumour model using lipoplex containing Gd3^+^ chelates [25].

These experiments used normal healthy rats to evaluate the concepts but future studies could use orthotopic brain tumour models, correlating tumour targeted gene delivery with MRI analysis. Gadolinium contrast agents based on the macrocyclic gadolinium chelator, DOTA could be used in future studies in the same LPD format which would be far more kinetically stable than acyclic gadolinium chelators, making them more persistent for imaging purposes and less toxic *in vivo* [26]. However, the DTPA reagent used here was adequate for proof of concept studies.

### 4.5 LA-ICP-MS analysis for MRI validation

Unexpectedly, a strong MRI signal was detected in the 6 h control brain, although it had been treated with a Gd-free LPD formulation. Histological analysis of sections of this brain revealed a small degree of haemorrhage in the vicinity of the MRI signal, close to the cannula insertion site, suggesting the possibility of a contrast signal arising from iron from blood products released in the haemorrhage site. To address this inconsistency in the MRI data, brain tissue sections from all animals, particularly regions associated with contrast effects, were analysed by LA-ICP-MS, a powerful technique for imaging of the distribution and relative concentration of specific metal ions in tissue sections. This technique provides a higher degree of resolution and sensitivity than MRI and an independent means of validating the MRI data [27]. Analysis revealed that all hyperintense MRI signals in the Gd^3 +^-nanocomplex treated animals at 6 h, and 48 h were associated solely with ^157^Gd atoms, while in the 24 h animal the signal on the right side of the brain was mainly from ^157^Gd but was also associated with paramagnetic iron (^57^Fe). In the 6 h control animal, which received no Gd^3 +^, there was a strong MRI signal but LA-ICP-MS analysis revealed that this signal was associated solely with ^57^Fe arising from haemorrhage at the site of injection. This study therefore provides a compelling example of the capacity of LA-ICP-MS to unambiguously differentiate different metal ions as sources of MRI contrast effects, such as iron-associated haemorrhage, allowing sensitive validation of MR images [16].

### 4.6 Histological analysis of *in vivo* transfection

Although the targeted Gd-LPD nanocomplex formulation was optimised for transfection of U87-MG glioblastoma cells, *in vivo* it demonstrated efficient GFP transfection in rat brain of ED-1-positive microglial cells. This observation itself opens up the possibility of developing gene therapy treatment for gliomas since glioma tissue consists of up to 30% of microglial cells which contribute to tumour cell invasion [28] making them potential targets for gene-based therapeutics. Future studies using orthotopic models of brain tumours will explore this possibility further as well as targeted transfection of the tumour itself. The fact that no transfection was observed in neurons stained with NeuN antibodies (data not shown) suggests the possibility of delivering therapeutics specifically to tumour-associated cells. Other diseases that may be treated by microglial-targeted gene therapies include lysosomal storage diseases, such as metachromatic leukodystrophy (MLD) caused by deficiency of the enzyme for arylsulfatase A [29].

### 4.7 Implications for future studies

Future studies will aim to improve the *in vivo* performance of the LPD formulation in terms of its transfection and targeting efficiency, and to increase the extent of dispersal which will require modulating the biophysical properties of the nanocomplexes. For example, while the LPD size distribution and cationic charge is typical for self-assembling electrostatic complexes it is not optimal for maximal distribution in the brain by CED where it has been shown that anionic or neutral particles distributed much better than cationic particles [6]. A similar problem of poor distribution was observed with a cationic HIV TAT peptide modified DNA nanolipoparticle administered to rat brain by CED [7]. Further, a size of 80 nm or less gave much better distribution than particles of 200 nm. Thus, while the majority of LPD nanoparticles produced (30–100 nm) are within the proposed optimal size range their cationic charge is probably restricting the extent of their distribution in brain by CED and future studies will aim to produce neutral or anionic LPD nanocomplexes with a narrower size distribution range.

Combining contrast agents with nanoparticle gene therapy formulations could offer new modalities for the non-invasive, real time monitoring of therapeutic gene delivery formulations, for example changes in their localisation over time in relation to targets sites, persistence. MRI is also useful for evaluating the outcome of a CED procedure, which can be highly dependent on cannula localisation and possible reflux along the needle track. Such formulations could lead to the development of new therapeutics for diseases of the brain including cancers and neurodegenerative diseases.

## Figures and Tables

**Table 1:** Liposome formulations prepared (weight ratios).

	DOTAP	DOPE	Gd lipid	% Gd lipid
DOTAP/DOPE	1	1	0	0
MW5	0.8	1	0.2	10
MW6	0.7	1	0.3	15
MW7	0.6	1	0.4	20

**Table 2:** Particle sizing and surface charge of nanoparticles in water.

Sample	Particle size (nm)	PDI	Zeta PD (+ mV)
*Liposomes (L)*			
DOTAP/DOPE	68.0 ± 1.0	0.21	27.4 ± 4.3
MW5	63.5 ± 0.6	0.23	49.2 ± 0.8
MW6	69.7 ± 0.5	0.23	27.6 ± 1.8
MW7	58.5 ± 0.4	0.21	24.6 ± 2.6

*Lipid/peptide/DNA (LPD)*			
DOTAP/DOPE	58.2 ± 0.7	0.13	36.0 ± 0.7
MW5	59.3 ± 0.5	0.08	73.9 ± 2.1
MW6	61 ± 0.5	0.11	44.1 ± 3.5
MW7	71.6 ± 0.7	0.24	20.2 ± 1.8

L = sonicated cationic liposome all formulated in a 1:1 ratio with DOPE. LPD = Lipid + peptide + plasmid DNA nanocomplex (1:4:1 weight ratio). PDI = polydispersity index.
